# Mineral accumulation in vegetative and reproductive tissues during seed development in *Medicago truncatula*

**DOI:** 10.3389/fpls.2015.00622

**Published:** 2015-08-14

**Authors:** Christina B. Garcia, Michael A. Grusak

**Affiliations:** Department of Pediatrics, United States Department of Agriculture/Agricultural Research Service Children's Nutrition Research Center, Baylor College of MedicineHouston, TX, USA

**Keywords:** legume, minerals, nutrition, partitioning, seeds

## Abstract

Enhancing nutrient density in legume seeds is one of several strategies being explored to improve the nutritional quality of the food supply. In order to develop crop varieties with increased seed mineral concentration, a more detailed understanding of mineral translocation within the plant is required. By studying mineral accumulation in different organs within genetically diverse members of the same species, it may be possible to identify variable traits that modulate seed mineral concentration. We utilized two ecotypes (A17 and DZA315.16) of the model legume, *Medicago truncatula*, to study dry mass and mineral accumulation in the leaves, pod walls, and seeds during reproductive development. The pod wall dry mass was significantly different between the two ecotypes beginning at 12 days after pollination, whereas there was no significant difference in the average dry mass of individual seeds between the two ecotypes at any time point. There were also no significant differences in leaf dry mass between ecotypes; however, we observed expansion of A17 leaves during the first 21 days of pod development, while DZA315.16 leaves did not display a significant increase in leaf area. Mineral profiling of the leaves, pod walls, and seeds highlighted differences in accumulation patterns among minerals within each tissue as well as genotypic differences with respect to individual minerals. Because there were differences in the average seed number per pod, the total seed mineral content per pod was generally higher in A17 than DZA315.16. In addition, mineral partitioning to the seeds tended to be higher in A17 pods. These data revealed that mineral retention within leaves and/or pod walls might attenuate mineral accumulation within the seeds. As a result, strategies to increase seed mineral content should include approaches that will enhance export from these tissues.

## Introduction

Legumes are an important food source worldwide. Because different species have the ability to grow in a variety of climates, legumes contribute to the staple food supply in many countries (Broughton et al., 2003; Vaz Patto et al., 2014). Improvements in the nutritional quality of these crops, therefore, will have far-reaching benefits (Vaz Patto et al., 2014). In particular, the goal of developing crops with higher micronutrient density has gained attention as an attractive strategy for combatting micronutrient malnutrition, a significant human health issue across the world (Murgia et al., 2012; Vaz Patto et al., 2014). Among legume species, seed micronutrient concentration is variable and higher relative to the concentration in cereals suggesting that the capacity for increasing nutrient concentration in legume seeds is greater (Blair, 2013).

Seed nutrient concentration is determined, in part, by source tissue function. It is well-established that adjacent leaves provide the majority of carbon- and nitrogen-containing molecules to developing seeds (reviewed in Weber et al., 2005; Munier-Jolain et al., 2008). Similarly, minerals are also supplied by the leaves. This has been described mostly in cereals with respect to micronutrient remobilization from flag leaves to developing grains. For example, in wheat, the amount of Fe and Zn translocated from the leaves was associated with differences in grain nutrient concentration (Uauy et al., 2006). In barley grown under nutrient-replete conditions, the leaves were the predominant source of Zn during early grain filling (Hegelund et al., 2012). Research in dicot species also suggest that mineral export from the leaves contributes to seed nutrient concentration. In pea, net remobilization of S, Cu, and Fe from the vegetative tissues (leaves and stems) was observed during seed mineral accumulation (Sankaran and Grusak, 2014). In another study, the expression of a putative vacuolar sulfate transporter *MtSULT4;1* was increased in the leaves of S-deficient *Medicago truncatula*, and this was associated with greater partitioning of S to the seeds and decreased retention of S in the leaves (Zuber et al., 2013). All together, these data indicate that the leaves act as a significant source of minerals for growing seeds.

The pod walls also act as a nutrient source for developing seeds. Twenty percent of the seed N content and between 9 and 40% of the mineral content was provided by the pod walls in pea (Schiltz et al., 2005; Sankaran and Grusak, 2014). In other legumes, mobilization of minerals from the pod wall accounted for 4–39% of the total seed mineral content (Hocking and Pate, 1977). From these studies, it is clear that, depending on the mineral, transport mechanisms within the pod wall significantly impact the seed content at maturity; however, very little is understood about the processes occurring within the pod wall. As a result, much remains to be characterized regarding the function of the pod walls as well as the leaves in relation to mineral export. Considering that both the leaves and pod walls supply nutrients to developing seeds, manipulation of the transport processes within both of these organs may increase nutrient allocation to the seeds (Bennett et al., 2011; Pottier et al., 2014).

Maximizing nutrient content in sink tissues by altering source tissue processes will require understanding the coordination between accumulation in sinks and export from source tissues (Pottier et al., 2014). Unfortunately, only a few studies have reported concurrent changes in leaf, pod wall, and seed mineral content. Early work with pea and lupin species concluded that different sources were responsible for contributing minerals through the progression of seed development in legumes (Hocking and Pate, 1977). Translocation from the leaves provided minerals during intermediate stages of seed development, while the pod wall and seed coat supplied minerals during the later stages of seed development (Hocking and Pate, 1977). This was supported by ^15^N-labeling experiments in pea, which demonstrated that the contribution of N from the leaves decreased during the latter stages of seed filling while the relative proportion of N remobilized from the pod walls increased (Schiltz et al., 2005). Aside from their role in nitrogen fixation, root nodules (which, like leaves, may be associated with senescence processes) have also been shown to provide minerals to developing seeds (Burton et al., 1998; Van de Velde et al., 2006). These studies have provided a foundation for defining source-sink relationships in legumes and indicate that a comprehensive understanding of these relationships will require additional knowledge about processes occurring in the leaves, pod walls, and seeds.

Genotypic differences in seed mineral concentration and content have been previously described in the model legume, *M. truncatula* (Sankaran et al., 2009). We sought to explore whether these disparities were due to variations in source tissue activities by characterizing the dry matter and mineral accumulation in the leaves, pod walls, and seeds of two different *M. truncatula* genotypes. We found that some minerals displayed similar patterns of accumulation and loss within separate organs and that alterations in mineral content coincided across different organs. Genotypic differences in growth and mineral concentration and content were also observed, particularly in the leaves and pod walls. These data allowed us to propose a model highlighting the contribution of different source tissues, particularly the pod walls, to seed mineral content at maturity.

## Methods

### Plant growth conditions

Seeds of *Medicago truncatula* ecotypes A17 Jemalong (A17) and DZA315.16 (DZA) were scarified in concentrated sulfuric acid for 2 min then rinsed with water (approximately 7x). Seeds were placed in moistened germination packets that were then placed in zip-top bags, left open, and vernalized at 4°C for 2 weeks. Seedlings were planted in a 2:1 Metro-Mix:vermiculite (Sun Gro Horticulture, Bellevue, WA) mixture at a density of 1–3 plants per pot (1 plant per pot for seed and pod wall growth dynamics experiments, 1 or 2 plants per pot for DZA leaf area analysis, 3 plants per pot for mineral accumulation experiments and A17 leaf area analysis) and grown in a greenhouse located at the Children's Nutrition Research Center in Houston, TX for the duration of each experiment. For the developmental time course, plants were grown from August to November 2012 and from April to August 2013 (including two replicates for experiments on reproductive tissue growth dynamics). Plants for leaf area analyses were grown from March to June 2014. Nutrient solution (1.0 mM KNO_3_, 0.4 mM Ca(NO_3_)_2_, 0.15 mM KH_2_PO_4_, 0.1 mM MgSO_4_, 25 μM CaCl_2_, 25 μM H_2_BO_3_, 1 μM Fe(III)EDDHA, 2.0 μM MnSO_4_, 2.0 μM ZnSO_4_, 0.5 μM CuSO_4_, 0.5 μM H_2_MoO_4_, 0.1 μM NiSO_4_) was delivered by an automatic irrigation system three times daily (Sankaran et al., 2009).

### Harvest and mineral analysis

Leaves and pods were harvested at pollination (day 0), 4, 8, 12, 16, 20, 24, 28, 32, and 36 days after pollination (DAP), and at pod maturity (pod abscission). The seeds were manually removed from the pods, all tissues were incubated at 65°C to dryness and reserved for acid digestion or tissue growth analysis. For acid digestion (Farnham et al., 2011), samples were treated with nitric acid (HNO_3_) overnight then incubated at 125°C for 2.5 h. Thirty percent (30%) hydrogen peroxide (H_2_O_2_) was added to the sample solutions followed by a 1 h incubation at 125°C (2x). The samples were then incubated at 200°C to dryness. After cooling, the samples were resuspended in 2% HNO_3_ and analyzed by inductively coupled plasma optical emission spectroscopy (ICP-OES, CIROS ICP model FCE12; Spectro, Kleve, Germany) for determination of Ca, Cu, Fe, K, Mg, Mn, Mo, P, S, and Zn concentration. The ICP-OES was calibrated daily with certified standards. Tomato leaf control and blank samples were included with each sample set to ensure that the instrument calibration remained consistent.

### Leaf area analysis

Flowers were tagged on the day of pollination (day 0), and an image of the subtending leaf was taken by placing the leaf on top of photographic paper, covering the leaf and paper with a thin piece of plexiglass, and exposing the paper to light. Leaf images were taken once every week for 4 weeks for A17 or 5 weeks for DZA. The final time point was determined by pod abscission. Images were scanned using a Canon CanoScan LiDE70 scanner, and leaf areas were determined using ImageJ (Rasband)^1^. The percent change of each leaf at a specific time point was calculated relative to the area at day 0, and the average percent change for each ecotype was determined with seven leaves from A17 and six leaves from DZA315.16.

### Statistical analyses

Statistical analyses were carried out using MatLab software R2014a/R2015a (Mathworks, Natick, MA). Repeated measures ANOVA was performed to determine statistically significant (*p* < 0.05) changes in mineral concentration, mineral content, and leaf area over time in datasets displaying equal variance. Friedman's test was performed on datasets displaying unequal variance to determine significant changes (*p* < 0.05) in these parameters. For analysis of Fe and Zn in the leaves and Fe in the pod walls of A17 plants, the data from 16 DAP (for leaves) and 8 DAP (for pod walls) were omitted in statistical analyses due to sample contamination.

**Figure 1:**
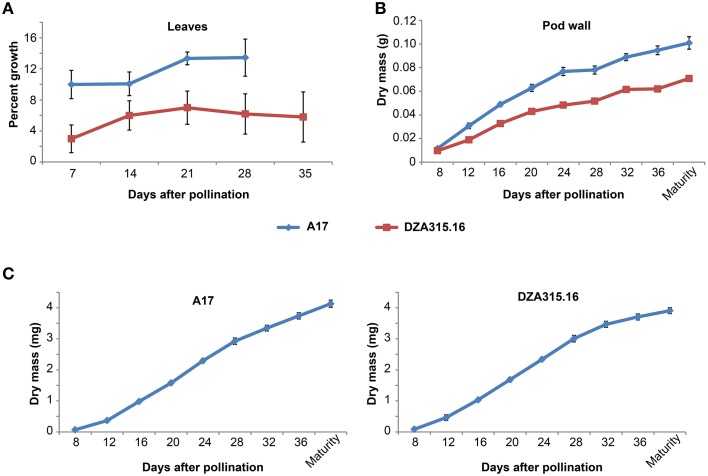
**Vegetative and reproductive tissue growth dynamics. (A)** Percent growth of leaflets for A17 (blue line) and DZA315.16 (red line) was calculated relative to the total leaflet area at pollination. The percent growth of leaflets from seven leaves for A17 and six leaves for DZA315.16 was averaged for each time. **(B,C)** Dry masses of pod walls **(B)** and seeds **(C)** were averaged from two replicated experiments. Tissues were harvested at 4-day intervals during pod development beginning at 8 days after pollination and continuing through pod maturity for ecotype A17 (blue line) and DZA315.16 (red line). For all panels, error bars display SEM.

Pod wall and seed growth datasets were unbalanced, so smaller test sets were generated from the original dataset by random sampling to use for statistical analyses. Friedman's test was performed on 3 separately generated test sets for each ecotype-tissue combination, and the change in mass over time was considered significant if all 3 test sets reached significance levels (*p* < 0.05). Wilcoxon signed rank tests were used for *post-hoc* pairwise comparisons to determine significant differences (*p* < 0.001, α = 0.05 with Bonferroni correction) between time points. All pod wall mineral content data were extrapolated using pod wall mass measurements shown in Figure 1. DZA seed mineral content data from plants grown in 2012 were also extrapolated; seed mass measurements shown in **Figure 4** were used to calculate content at days 20 and 24. The average masses of simultaneously grown, undigested seeds were used to calculate content at the remaining time points.

Ten mature pods from two separate plantings (for a total of 20 pods) were harvested from both ecotypes, and the number of seeds per pod was counted and averaged. To calculate the seed mineral content per pod, the individual measurements of seed content at maturity were multiplied by the average number of seeds per pod, then averaged. Two-tailed *t*-tests were used to calculate significant differences (*p* < 0.005, α = 0.05 with Bonferroni correction) between ecotypes.

For pairwise comparisons of mineral data between ecotypes, Mann-Whitney *U*-tests (for data with unequal variance) or two-tailed, unpaired *t*-tests (for data with equal variance) were used to calculate *p*-values. Positive false discovery rates (pFDR) were calculated for all *p*-values, and differences were considered significant if pFDR ≤ 0.05.

For leaf area measurements, two images of each leaf were analyzed at each time point, and the areas of the two images were averaged. Repeated measures ANOVA or Friedman's test was performed as described above to determine changes in leaf area over time.

The log_2_ fold differences between the average mineral concentration at a given time point and the average concentration at the first measured time point was calculated for each tissue. Hierarchical clustering was performed using Cluster 3.0 (de Hoon et al., 2004) using Spearman rank correlation and average linkage. Data were visualized using Java Treeview (Saldanha, 2004).

## Results

### Differences in leaf mineral concentration are few but distinct between A17 and DZA315.16

Previous studies reported variations in seed mineral concentration and content between the ecotypes A17 and DZA315.16 (Sankaran et al., 2009). In order to identify putative control points underlying these differences, we studied overall changes in mineral concentration and content in the leaves, pod walls, and seeds during pod development. The concentrations of approximately half of the minerals in the leaves of either genotype did not change significantly during pod development (Ca, Fe, K, Mo, P, and S in A17 and Fe, Mn, Mo, P, and Zn in DZA315.16); however, the Cu concentrations tended to decrease and the Mg concentrations tended to increase in the leaves of both ecotypes. Additionally, other minerals, such as Mn and Zn, also appeared to decrease in A17, while Ca, K, and S displayed an increasing trend in DZA leaves (Supplementary Table 1).

Since mineral concentration depends on mass and is subject to change depending on the growth of the organ, we also studied the growth dynamics of the leaves by calculating changes in leaf area over time. A17 leaf area increased by approximately 10% within the first week of pod development and further increased between 14 and 21 DAP (Figure 1A). The area of DZA leaves also tended to increase over time; however, the changes in percent growth were not statistically significant (Figure 1A). Average leaf dry mass displayed similar trends for both ecotypes, but the differences in mass for A17 were not statistically significant (Supplementary Presentation 1). It should be noted, however, that measuring leaf dry matter growth required sequential leaf harvests from the plant and because leaf size was variable, even among those leaves harvested from the same branch, it appears that measuring average leaf mass was not as sensitive as measuring area on the same leaves. Taken together, these data indicate that leaf expansion continued through pollination and the initial stages of pod development in A17, while the size of DZA leaves remained constant.

In order to determine whether the changes in concentration were the result of tissue growth or net changes in content, we extrapolated mineral content based on average leaf dry mass at each time point (Supplementary Presentation 1). Mineral content changed significantly for seven of 10 measured minerals in each ecotype, although the sets of minerals that changed were not identical between genotypes. In A17 plants, the total content of Ca, K, Mg, Mn, Mo, and S tended to increase during reproductive development, and similarly, Ca, K, Mg, and S increased in DZA leaves (Table 1, Supplementary Presentation 2). The Fe content also increased in DZA plants, but was not significantly different over time in the leaves of A17 plants (Table 1, Supplementary Presentation 2). In contrast, the Cu content decreased in DZA leaves (Table 1, Supplementary Presentation 2). The Cu content in A17 leaves was also significantly altered over time; however, comparison of the contents at pollination and maturity did not appear significantly different from one another (Table 1), indicating that differences in content occurred at other points during the time course. In the same way, there did not appear to be a significant difference between the Zn content in DZA leaves at pollination and maturity, although the statistics indicated that there were differences in Zn content over the course of pod development (Table 1).

**Table 1:** **Leaf mineral content at pollination and pod maturity**.

**Mineral**	**A17**			**DZA**			**A vs. D**	
	**Overall**	**Pollination**	**Maturity**	**Overall**	**Pollination**	**Maturity**	***P***	***M***
Ca (μg/leaf)	+	131±5	400±25	+	191±10	496±8	+	−
Cu (ng/leaf)	+	63.7±4.1	66.8±8.9	+	113±4	76.3±8.9	+	−
Fe (ng/leaf)	−	658±116	976±58.2	+	901±57	1390±224	−	−
K (μg/leaf)	+	212±10	497±38	+	268±15	407±7	−	−
Mg (μg/leaf)	+	53.5±2.8	152±8	+	64.0±3.9	155±6	−	−
Mn (ng/leaf)	+	124±8	180±20	−	385±21	401±55	−	+
Mo (ng/leaf)	+	306±78	983±392	−	219±74	415±126	−	−
P (μg/leaf)	−	46.0±3.1	62.4±4.6	−	59.9±6.7	67.6±5.9	−	−
S (μg/leaf)	+	30.5±2.8	59.6±4.0	+	39.4±2.0	70.8±5.7	−	−
Zn (ng/leaf)	+	138±11	153±17	+	263±10	272±2	+	+

*Results from a priori statistical tests (overall), average leaf mineral content at pollination and pod maturity (Maturity), and results of pairwise comparisons between A17 and DZA315.16 contents at pollination (P) and maturity (M) are given. Mineral content was extrapolated based on concentrations summarized in Supplementary Table 1 and average leaf mass measurements of independently grown plants. Repeated measures ANOVA or Friedman's test were used to determine if there were differences in average mineral content at any time between pollination and maturity (overall). Minerals whose content changed significantly (p < 0.05) at any point during the time course are marked with a plus (+) sign; minerals whose concentration did not change significantly over time (p > 0.05) are marked with a minus (−) sign. Average content ± standard error of the mean (SEM) of four samples is given. For between ecotype analyses (A vs. D), pairwise comparisons (two-tailed t-test or Mann-Whitney U-test) were made between the contents at each time point. The positive false discovery rate (pFDR) was calculated for each p-value, and comparisons with pFDR < 0.05 were considered significant (+)*.

**Figure 2:**
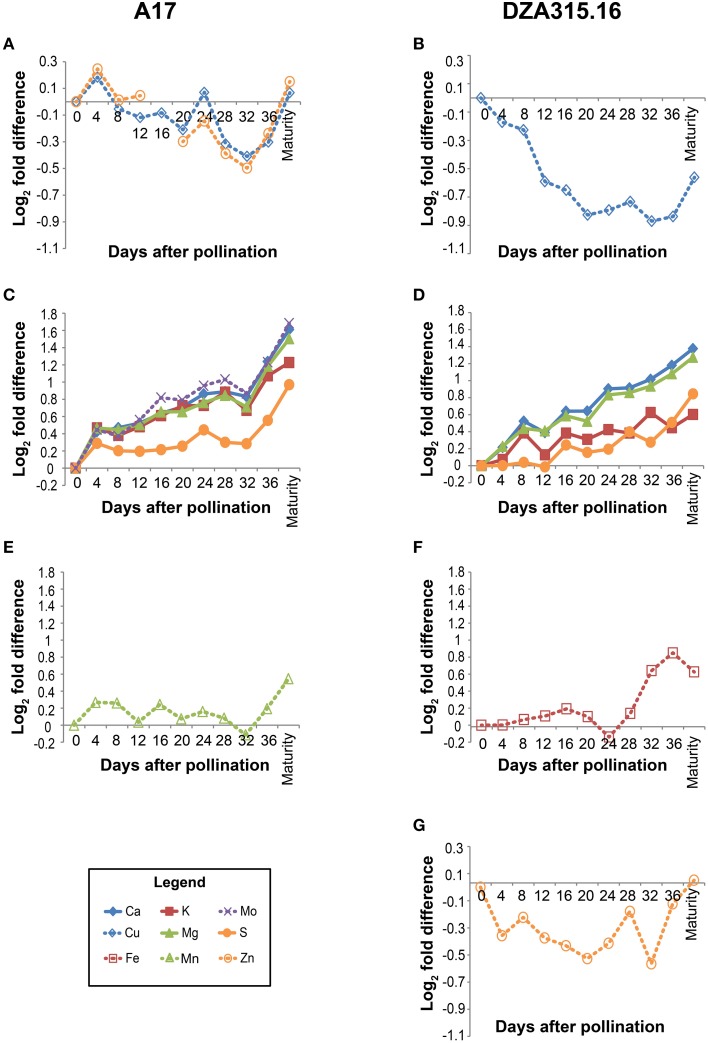
**Changes in leaf mineral content over time**. Fold differences between the average content at a given time point and the content at 0 days after pollination (DAP) were calculated. Log_2_ transformed fold differences were used as input for hierarchical clustering to identify groups of minerals displaying similar changes in content over time. Line graphs display the fold change in content at each time point (days after pollination) relative to the content at 0 DAP. Minerals that clustered together with correlation >0.8 in A17 (left column) and DZA (right column) are depicted in the same line graph. Minerals are represented by line color and style as follows: solid blue line, Ca, solid red line, K, solid green line, Mg, solid orange line, S, dashed blue line, Cu, dashed red line, Fe, dashed green line, Mn, dashed purple line, Mo, and dashed orange line, Zn. **(A)** Fold differences in Cu and Zn content in A17 leaves. Zn content at 16 DAP was omitted from statistical analyses due to possible sample contamination. **(B)** Fold differences in Cu content in DZA leaves. **(C)** Fold differences in Ca, K, Mg, S, and Mo content in A17 leaves. **(D)** Fold differences in Ca, K, Mg, and S content in DZA leaves. **(E)** Fold differences in Mn content in A17 leaves. **(F)** Fold difference in Fe content in DZA leaves. **(G)** Fold differences in Zn content in DZA leaves.

In order to more specifically define the patterns in content change over time and to determine whether there were similarities in those patterns among the minerals, we calculated the fold difference in content at each time point, relative to the content at 0 days after pollination (0 DAP). We also performed hierarchical clustering based on fold changes (Supplementary Presentation 3). In general, the macronutrients and micronutrients formed separate clusters (Figure 2). The exception to this observation was Mo, which clustered with the macronutrients in A17 leaves (Figure 2C). Furthermore, while there were differences in mineral accumulation and loss during earlier time points, almost all minerals displayed an increase in content between 32 DAP and pod maturity (Figure 2) revealing a net gain of nutrients during the latter stages of pod development. As a result, clusters could be distinguished by the changes in content during earlier stages of the time course. For example, between 0 and 32 DAP in A17 leaves, the Cu and Zn contents decreased (Figure 2A), the contents of Ca, K, Mg, S, and Mo increased (Figure 2C), and the Mn content remained relatively stable (Figure 2E). Similarly, in DZA leaves, the Cu and Zn contents decreased (Figures 2B,G), the macronutrient contents increased (Figure 2D), and the Fe content was constant through the beginning of pod development (Figure 2F). In all, these data suggest that the accumulation and loss of certain minerals was co-regulated.

Leaf mineral contents were compared at each time point to determine if there were differences between ecotypes. At pollination, the Ca, Cu, and Zn contents were significantly higher in DZA leaves compared with A17 leaves. The Cu and Zn contents in DZA were nearly double that of A17, while the Ca content in A17 leaves was approximately 70% of the DZA content (Table 1). Over time, the differences in Ca and Cu contents diminished so that by maturity, there were no significant differences in content of these minerals (Table 1, Supplementary Presentation 2). Zinc, in contrast, remained higher in DZA leaves throughout pod development, and the content was significantly higher at maturity as well (Table 1). In addition, the Mn content was significantly higher at maturity in DZA leaves, with DZA containing more than twice the content in A17 leaves (Table 1). Taken together, these data reveal genotype-specific differences in mineral storage in the leaves.

### Increases in mineral content were similar in the pod walls of both ecotypes

We also studied mineral accumulation and dry matter growth in pod walls throughout their reproductive development. The growth of A17 pod walls appeared to occur in two distinct phases: the first occurring from 8 DAP through 24 DAP and the second occurring from 24 DAP through maturity. The rate of growth appeared higher during the first phase compared to the second phase, and overall, the mass of pod walls at maturity was 9 times the value of the mass at 8 DAP (Figure 1B). DZA pod walls also displayed a steady increase during development, although there was little, if any, difference in growth rates between early and late development. Overall, the mass of DZA pod walls increased by 6 times from 8 DAP to maturity (Figure 1B). Comparing the two ecotypes revealed that there was a significant difference in mass starting at 12 DAP, and this difference persisted through maturity (Figure 1B).

**Figure 3:**
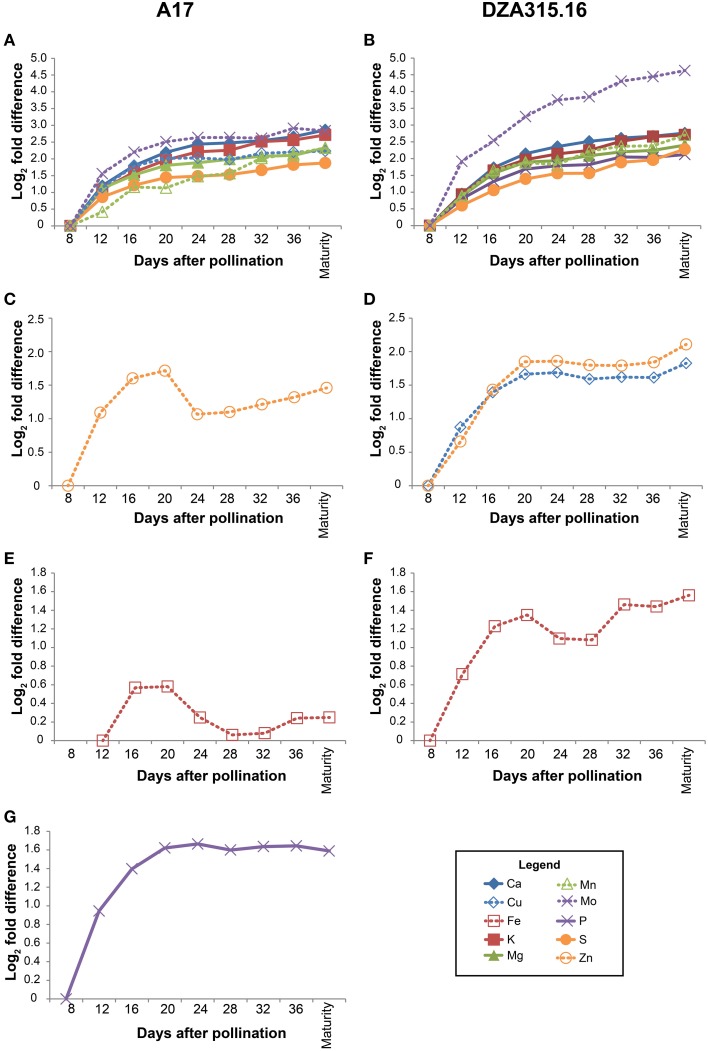
**Changes in pod wall mineral content over time**. Fold differences between the average content at a given time point and the content at 8 days after pollination (DAP) or 12 DAP (for Fe content in A17 pod walls) were calculated. Log_2_ transformed fold differences were used as input for hierarchical clustering to identify groups of minerals displaying similar changes in content over time. Line graphs display the fold change in content at each time point (days after pollination) relative to the content at 8 DAP or 12 DAP (for Fe in A17 only, **C**). Minerals that clustered together with correlation >0.8 in A17 (left column) and DZA (right column) are depicted in the same line graph. Minerals are represented by line color and style as follows: solid blue line, Ca, solid red line, K, solid green line, Mg, solid purple line, P, solid orange line, S, dashed blue line, Cu, dashed red line, Fe, dashed green line, Mn, dashed purple line, Mo, and dashed orange line, Zn. **(A)** Fold differences in Ca, K, Mg, S, Cu, Mn, and Mo content in A17 pod walls. **(B)** Fold differences in Ca, K, Mg, P, S, Mn, and Mo content in DZA pod walls. **(C)** Fold differences in Zn content in A17 pod walls. **(D)** Fold differences in Cu and Zn content in DZA pod walls. **(E)** Fold difference in Fe content in A17 pod walls. **(F)** Fold difference in Fe content in DZA pod walls. **(G)** Fold difference in P content in A17 pod walls.

Using the growth data, the pod wall mineral contents were extrapolated from concentration measurements (summarized in Supplementary Table 2). While the concentration decreased for most minerals in the pod walls during reproductive development (Supplementary Table 2), analysis of the change in mineral content over time revealed that there was net accumulation of minerals (Table 2, Supplementary Presentation 4). Hierarchical clustering was performed to identify minerals that displayed similar patterns of content change. This generated four distinct groups of minerals in A17 and one group in DZA (Supplementary Presentation 5). For almost all minerals, the greatest amount of accumulation occurred between 8 and 20 DAP (Figure 3, Supplementary Presentation 4). After 20 DAP in A17, Ca, K, Mg, S, Cu, Mn, and Mo (Figure 3A) tended to increase. The P content in A17 also displayed a similar pattern but was more stable from 20 DAP through pod maturity (Figure 3F). Similar to the cluster depicted in Figure 3A, the minerals in DZA pod walls also tended to increase after 20 DAP (Figures 3B,D). The micronutrients displayed some variability in pattern between 20 and 32 DAP, but after 32 DAP, the content of all these minerals increased (Figure 3D). Zinc and Fe in A17 pod walls were separated from the other minerals by clustering analysis and were distinguished by a decrease between 20 and 24 DAP or 28 DAP, respectively (Figures 3C,E).

**Table 2:** **Pod wall mineral content**.

**Minerals**	**A17**			**DZA**			**A vs. D**	
	**Overall**	**8 DAP**	**Maturity**	**Overall**	**8 DAP**	**Maturity**	**8**	***M***
Ca (μg/pod wall)	+	155±17	1130±69	+	197±6	1340±109	−	−
Cu (ng/pod wall)	+	71.2±3.4	334±4	+	120±3	424±6	+	+
Fe (ng/pod wall)	−	^*^ 768±194	913±91	+	696±98	2050±478	−	−
K (μg/pod wall)	+	195±15	1280±13	+	193±6	1260±113	−	−
Mg μg/pod wall)	+	84.6±8.6	425±49	+	92.5±1.4	488±21	−	−
Mn (ng/pod wall)	+	212±12	1060±66	+	478±30	3110±323	+	+
Mo (ng/pod wall)	+	137±30	976±170	+	71.8±19.0	1770±192	−	−
P (μg/pod wall)	+	49.3±2.4	148±14	+	49.7±2.1	216±26	−	−
S (μg/pod wall)	+	21.2±1.0	78.1±10.3	+	20.2±0.3	98.0±1.2	−	−
Zn (ng/pod wall)	+	351±51	963±313	+	436±31	1880±109	−	−

*Results from a priori statistical tests (overall), average pod wall mineral content at 8 days after pollination (8 DAP) and pod maturity (Maturity), and results of pairwise comparisons between A17 and DZA315.16 contents at 8 DAP (8) and maturity (M) are given. Mineral content was extrapolated from pod wall mineral concentrations and pod wall mass measurements summarized in Supplementary Table 2 and Figure 1, respectively, for each time point shown in Figure 1. Repeated measures ANOVA or Friedman's test were used to determine if there were differences in average mineral content at any time between 8 DAP and maturity (overall). Minerals whose content changed significantly (p < 0.05) at any point during the time course are marked with a plus (+) sign; minerals whose concentration did not change significantly over time (p > 0.05) are marked with a minus (−) sign. Average content ± standard error of the mean (SEM) of four samples is given. For between ecotype analyses (A vs. D), pairwise comparisons (two-tailed t-test or Mann-Whitney U-test) were made between the contents at each time point. The positive false discovery rate (pFDR) was calculated for each p-value, and comparisons with pFDR < 0.05 were considered significant (+)*.

Comparisons were also made between ecotypes for differences in pod wall mineral content. This revealed variation in only two minerals, Cu and Mn. For both nutrients, the content in DZA pod walls was approximately 2 times the content in A17 pod walls at 8 DAP. Differences in content of these minerals were also observed at maturity, when the Cu content was 0.3 times higher and the Mn content was 1.9 times higher in DZA pod walls than in A17 (Table 2, Supplementary Presentation 4). In summary, minerals accumulated in the pod walls primarily during the first 20 days of development and subsequently, showed modest variations in change. In addition, there were few differences between ecotypes in terms of mineral content, in spite of significant differences in the overall mass of the pod wall.

### Mineral accumulation and growth in the seeds were similar between ecotypes

To gain a clearer understanding of the relationship between seed growth and mineral accumulation, we also measured changes in individual seed mass and mineral content over time. The growth curves for seeds of both ecotypes appeared slightly sigmoidal with an inflection point at 24 DAP, and the seed mass increased by approximately 45 and 60 times from 8 DAP to maturity in DZA and A17 seeds, respectively (Figure 1C). In spite of the differences in the magnitude of change, there was no significant difference in individual seed mass between the two ecotypes at maturity.

Seed mineral content and concentration were determined during the latter stages of development (from 20 DAP through maturity). Approximately half of the minerals in each ecotype displayed changes in concentration from 20 DAP to maturity with Ca and Mn concentrations decreasing, while the concentrations of other minerals tended to increase (Supplementary Table 3). Nevertheless, the total Mn content in both A17 and DZA and the total Ca content in A17 seeds increased (Table 3, Supplementary Presentation 6), highlighting the general tendency of all minerals to increase in content. The exceptions to this trend were contents of Mg and P in A17 seeds, and Ca in DZA seeds, which did not alter significantly during this period of time (Table 3).

**Table 3:** **Seed mineral content**.

**Mineral**	**A17**			**DZA315.16**			**A vs. D**	
	**Overall**	**20 DAP**	**Maturity**	**Overall**	**20 DAP**	**Maturity**	**20**	**M**
Ca (μg/seed)	+	3.19±0.06	5.61±0.46	−	4.25±0.18	4.93±0.31	+	−
Cu (ng/seed)	+	14.1±0.9	44.7±1.4	+	29.6±5.5	94.9±3.3	−	+
Fe (ng/seed)	+	154±12	393±7	+	224±57	532±69	−	−
K (μg/seed)	+	21.5±0.7	43.4±1.5	+	24.4±2.0	41.8±0.5	−	−
Mg (μg/seed)	−	5.01±0.32	11.8±0.4	+	5.46±0.45	10.9±0.2	−	−
Mn (ng/seed)	+	37.1±1.3	73.5±6.1	+	42.5±3.6	70.6±1.2	−	−
Mo (ng/seed)	+	61.5±0.9	243±11	+	40.5±0.7	154±7	+	+
P (μg/seed)	−	11.7±0.4	36.8±1.2	+	10.3±1.1	35.6±0.6	−	−
S (μg/seed)	+	4.90±0.40	14.1±0.3	+	4.75±0.74	12.6±0.2	−	+
Zn (ng/seed)	+	90.8±2.7	220±19	+	115±17	257±24	−	−

*Results from a priori statistical tests (overall), average seed mineral content at 20 days after pollination (20 DAP) and pod maturity (Maturity), and results of pairwise comparisons between A17 and DZA315.16 contents at 20 DAP (20) and maturity (M) are given. For some DZA315.16 values, mineral content was extrapolated based on concentrations summarized in Supplementary Table 3 and seed mass measurements. Repeated measures ANOVA or Friedman's test were used to determine if there were differences in average mineral content at any time between 20 DAP and maturity (overall). Minerals whose content changed significantly (p < 0.05) at any point during the time course are marked with a plus (+) sign; minerals whose concentration did not change significantly over time (p > 0.05) are marked with a minus (−) sign. Average content ± standard error of the mean (SEM) of four samples is given. For between-ecotype analyses (A vs. D), pairwise comparisons (two-tailed t-test or Mann-Whitney U-test) were made between the contents at each time point. The positive false discovery rate (pFDR) was calculated for each p-value, and comparisons with pFDR < 0.05 were considered significant (+)*.

**Figure 4:**
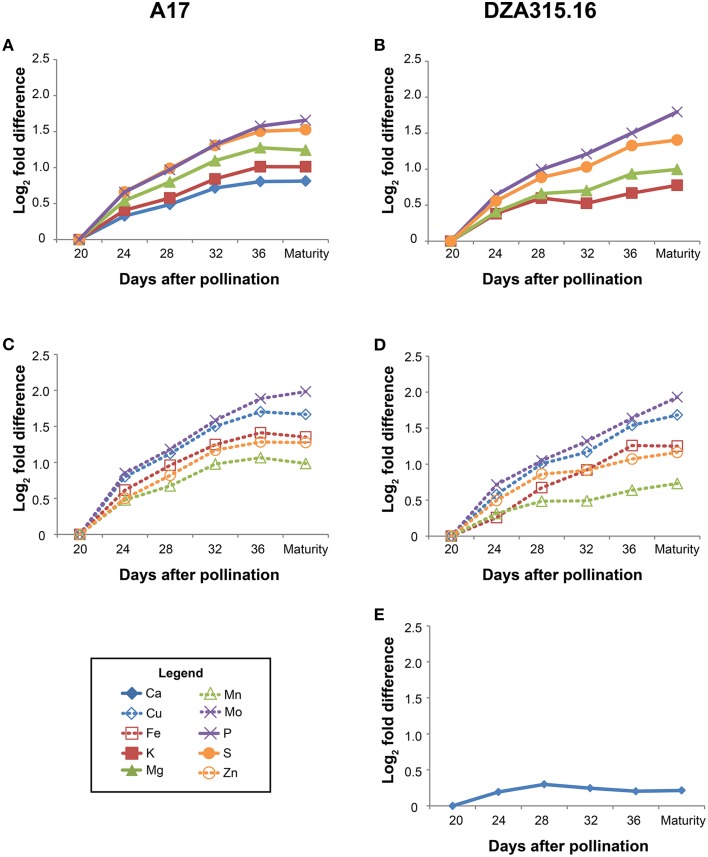
**Changes in seed mineral content over time**. Fold differences between the average content at a given time point and the content at 20 days after pollination (DAP) were calculated. Log_2_ transformed fold differences were used as input for hierarchical clustering to identify groups of minerals displaying similar changes in content over time. Line graphs display the fold change in content at each time point (days after pollination) relative to the content at 20 DAP. All minerals in A17 seeds clustered together (correlation > 0.8), but are depicted in separate panels (**A**, macronutrients, and **C**, micronutrients) to enhance visualization. Similarly, all minerals except Ca **(E)** clustered together (correlation > 0.8) in DZA seeds, but are depicted in separate line graphs (**B**, macronutrients, and **D**, micronutrients) for visualization purposes. Minerals are represented by line color and style as follows: solid blue line, Ca, solid red line, K, solid green line, Mg, solid purple line, P, solid orange line, S, dashed blue line, Cu, dashed red line, Fe, dashed green line, Mn, dashed purple line, Mo, and dashed orange line, Zn.

Those minerals that displayed significant changes in content over time were further categorized through hierarchical clustering (Supplementary Presentation 7). The patterns of accumulation were highly correlated in A17 seeds for all minerals, and similarly, all minerals except Ca clustered together in DZA seeds (Figure 4). The Ca content in DZA seeds increased minimally between 20 and 28 DAP and remained stable from 28 DAP through maturity (Figure 4E). Likewise, K, Mg, Mn, and Zn contents increased only slightly after 28 DAP (Figures 4B,D). In contrast, the other minerals accumulated through later stages of seed development. In A17 seeds, accumulation stabilized at 32 DAP, whereas in DZA seeds, the accumulation stabilized at 36 DAP for S, Mo, Cu, and Fe. Overall, both ecotypes displayed similar growth dynamics and patterns of mineral accretion in the seeds.

While there were similarities in overall trends of growth and mineral accumulation, there were a few differences between ecotypes with respect to individual seed mineral content. The Ca content was 0.3 times higher in DZA seeds than A17 seeds at 20 DAP, but the difference was not significant at maturity (Table 3). In contrast, the S content was similar at 20 DAP, but was significantly higher in A17 seeds at maturity (Table 3). For Cu and Mo, the differences in content persisted from 20 DAP through pod maturity. The Cu content in DZA seeds was twice the value of the content in A17 seeds at both time points, whereas the Mo content was approximately 0.5 times higher in A17 seeds at both time points (Table 3). Together, these data indicate that while seed growth and mineral accumulation patterns in both ecotypes were similar, there were genotype-specific differences in total mineral deposition in the seeds.

**Figure 5:**
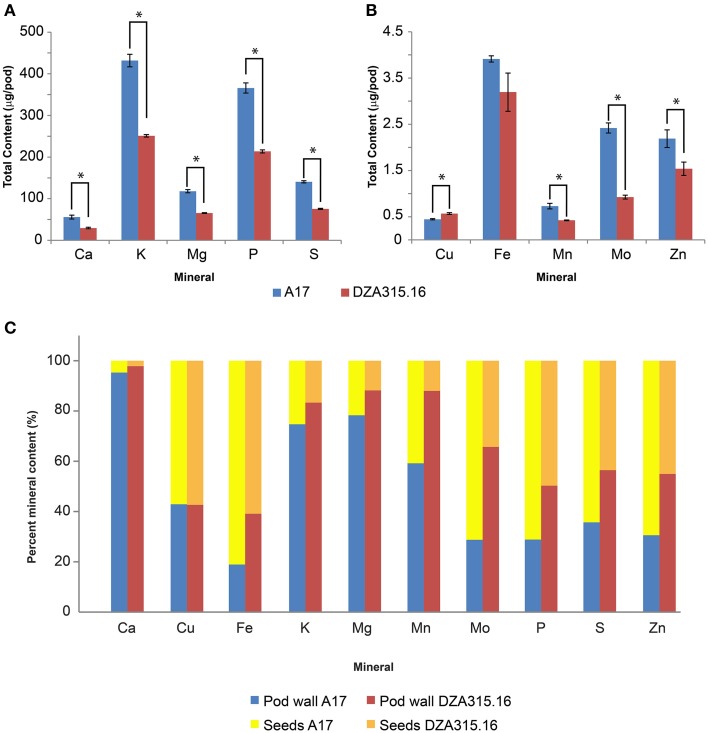
**Total seed mineral content and mineral partitioning per pod. (A,B)** display bar graphs depicting average seed mineral content in A17 pods (red bars) and DZA pods (blue bars). Error bars display SEM. Asterisks indicate significant differences (*p* < 0.05 with Bonferroni correction) between genotypes. This data is summarized in Table 4. The percentages of the total mineral content per pod contained in the pod walls and seeds were calculated and are presented in **(C)**. The relative distribution of each mineral between the pod walls (dark blue, A17, light blue DZA) and seeds (yellow, A17, brown, DZA) are shown.

Furthermore, due to genotypic differences in average seed number per pod, there were disparities in seed mineral content per pod. On average, A17 pods contained 9.95 seeds while DZA pods contained 6.00 seeds (data not shown). This resulted in higher seed mineral contents per pod in A17 for almost all minerals at maturity (Table 4, Figures 5A,B). The total macronutrient content of DZA seeds was 50–60% the total content of A17 seeds (Table 4, Figure 5A), while the differences between ecotypes were more variable with respect to the micronutrients. The total Mo content in DZA seeds was less than half of the total content in A17 seeds, whereas the Mn and Zn contents were approximately 60 and 70% of the A17 seed contents, respectively (Table 4 and Figure 5B). The total Cu content was significantly higher in DZA seeds compared with A17, and the differences in total seed Fe content was not statistically different between the two genotypes (Table 4, Figure 5B). Overall, mineral partitioning to the seeds tended to be higher in A17 pods when compared to DZA (Figure 5C). These data indicate that, as a whole, A17 mobilized a greater amount of minerals to the total seed pool within a pod.

**Table 4:** **Total seed mineral content per pod at maturity**.

**Mineral**	**A17**	**DZA**	***p* < 0.05**
Ca (μg)	55.8±4.6	29.6±1.9	+
Cu (μg)	0.445±0.014	0.569±0.020	+
Fe (μg)	3.91±0.07	3.19±0.41	−
K (μg)	432±15	251±3	+
Mg (μg)	118±4	65.4±1.1	+
Mn (μg)	0.731±0.060	0.423±0.007	+
Mo (μg)	2.42±0.11	0.925±0.042	+
P (μg)	366±12	214±4	+
S (μg)	141±3	75.5±1.3	+
Zn (μg)	2.19±0.19	1.54±0.14	+

*Mineral contents per seed at maturity (summarized in Table 3) were multiplied by the average number of seeds per pod in each ecotype: 9.95 for A17 and 6.00 for DZA315.16 (DZA). The average ± the SEM of each mineral in both ecotypes are presented. Two-tailed Student's t-tests were used to determine statistical differences between the two ecotypes. Significantly different (p < 0.05 with Bonferroni correction) minerals are labeled with a plus (+) sign. Minerals that were not significantly different between the two genotypes are labeled with a minus (−) sign*.

## Discussion

In this study, we have characterized growth dynamics and mineral accumulation in the reproductive tissues and their supporting source leaves during pod development in two genotypes of *M. truncatula*. Recombinant inbred lines generated by crossing these genotypes, A17 and DZA315.16, displayed significant variation in seed mineral concentration and content in an earlier study (Sankaran et al., 2009), and we queried whether this could be explained by differences in the mineral accumulation and partitioning during reproductive growth in the parent lines.

Overall, there were few differences in mineral content per seed at maturity between the ecotypes (Table 3), but there were significant differences in the total seed content per pod (Table 4) suggesting that there were differences in the total mineral flux to the seeds. A17 pods accumulated higher levels of most minerals in the seeds as a whole, but Cu content was significantly higher in DZA seeds (content per seed and total seed content per pod) compared with A17 (Tables 3, 4). This may have been due to greater mobilization of Cu from DZA leaves. The Cu content in DZA leaves was higher than that of A17 leaves at 0 DAP but similar at maturity, demonstrating a net loss of Cu from DZA leaves, with Cu content unchanged in A17 leaves over time (Table 1, Figures 2A,B). Previous studies in *Arabidopsis* and rice showed that defects in Cu transport from leaves to seeds led to reductions in seed Cu concentrations (Waters et al., 2006; Zheng et al., 2012; Benatti et al., 2014); thus, the leaf and seed differences in Cu content between A17 and DZA may have arisen from higher levels of expression of one or more factors like metallothioneins or yellow stripe-like (YSL) transporter proteins in DZA leaves. Metallothioneins were previously shown to be involved in the senescence-related remobilization of Cu in *Arabidopsis* leaves, although their function appeared to be more relevant in the context of Cu deficiency (Benatti et al., 2014). Expression of YSL proteins in the leaves of *Arabidopsis* and rice has been associated with mobilization of Cu from leaves to developing seeds and grains (Waters et al., 2006; Zheng et al., 2012). These pathways, either working alone or in parallel, would contribute to greater Cu remobilization from leaves to seeds (Benatti et al., 2014). An opposing mechanism, in which transport of minerals from the pod wall to the seeds is restricted may also affect nutrient flux. This may have been the case with Mo, which was significantly higher in A17 seeds (Table 3). In DZA pod walls, Mo displayed a greater level of accumulation compared with A17 (Figures 3A,B). Taken together, these data suggest that the efficiency of mineral translocation from the leaves to the pods and from the pod wall to the seeds can influence the total mineral content in the seeds, at least when source organ mineral substrate pools are available.

Our studies also revealed coordination between development and changes in mineral content within each tissue. The subtending source leaves of A17 plants expanded during the first 3 weeks of pod growth (Figure 1A), indicating ongoing leaf development. During this time, the changes in mineral content were minimal, with some minerals displaying slight tendencies toward loss (Figure 2A) or accumulation (Figure 2B); however, the most substantial changes in leaf mineral content, for Cu and Zn in particular, did not occur until after leaf expansion had subsided (Figures 2A,C,E). In contrast, the leaf area was not significantly altered in DZA plants during pod development (Figure 1A), and in the first 20 days both the Cu and Zn content decreased to minimal levels in DZA leaves (Figures 2F,G). Because decreased micronutrient content has been associated with leaf senescence, the terminal stage of leaf development (Himelblau and Amasino, 2001; Marschner, 2012), these data suggest that within the first 20 days of pod development, the senescence of subtending leaves had initiated and progressed in DZA. Overall, this highlights genotypic differences in leaf development that could modulate seed mineral content.

Coordination of mineral dynamics and dry matter accumulation also displayed slight genotypic differences. In both ecotypes, the pod wall mineral content increased most rapidly between 8 and 20 DAP (Figure 3, Supplementary Presentation 4). Characterization of pod wall development revealed that the phloem in A17 pod walls had fully differentiated by 13 DAP (Wang and Grusak, 2005), so the gradual decline in the rate of mineral accumulation after 12 DAP may reflect increasing phloem-mediated mineral transport to developing seeds. The similarity in pod wall mineral accumulation between the two ecotypes during these early stages (i.e., between 8 and 20 DAP), suggests that this aspect of pod wall development is conserved. Beyond 20 DAP, the contents of most pod wall minerals changed minimally (Figure 3). As exceptions to this trend, Zn in A17 pod walls, and Fe in the pod walls of both ecotypes decreased between 20 and 24 DAP (Figures 3C,E). These changes in mineral content coincided with the change in the rate of dry matter acquisition in DZA pod walls (Figure 1B), but preceded a change in the rate of dry matter acquisition by 4 days in A17 pod walls (Figure 1B). These data indicate that there was coordinated regulation of mineral and dry matter accumulation in DZA pod walls and an uncoupling of these two phenomena in A17 pod walls.

In the seeds, there was also an association between dry matter and mineral accumulation. Dry mass increased in a linear fashion between 12 and 28 DAP in both ecotypes (Figure 1C) corresponding with the storage phase for *M. truncatula* (Gallardo et al., 2007). The mineral content also increased during this time. In our study, which focused on seed mineral accumulation starting at 20 DAP, the highest rate of accumulation occurred between 20 and 28 DAP; after 28 DAP, the slope declined and approached 0 at 32 DAP for most minerals (Figure 4). The timeframe between 20 and 24 DAP corresponds to the end of storage protein synthesis (Gallardo et al., 2007), suggesting that mineral accumulation was associated with amino acid availability. This may be due to the simultaneous liberation of amino acids and minerals by the process of senescence (Marschner, 2012). Indeed, earlier onset of senescence in leaves was associated with increased grain protein, Zn, and Fe content in wheat (Uauy et al., 2006). The data presented here suggest that senescence of A17 leaves occurs after 20 DAP, during which time nutrient flux through the subtending leaf was sufficient to support accumulation in the developing pods and seeds without a significant change in the steady state content of the leaves. It is recognized, however, that some minerals and amino acids may have been derived from older, senescing tissues. Future research utilizing isotopic labeling will distinguish between these possibilities.

**Figure 6:**
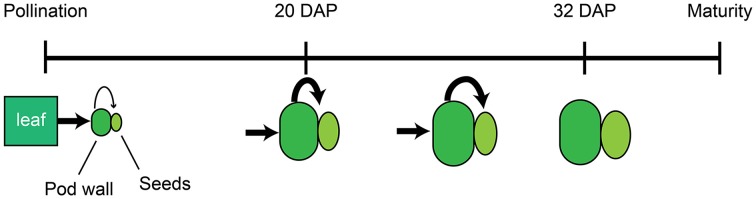
**Model of mineral accumulation in the pod walls and seeds during development**. Beginning at pollination, the subtending leaf (green rectangle) provides nutrients to developing pods, comprised by the pod wall (dark green oval), and seeds (light green ellipse). The pod wall delivers nutrients to the developing seeds. The relative amount of nutrient transport from source to sink tissues is indicated by the size of the black arrows. At 20 DAP, the pod wall and seeds have increased in size, and the relative amount of nutrient transport from the pod wall to the seeds has also increased. After 20 DAP, transport to the seeds remains at the same level, and growth of the pod wall and seeds continues. By 32 DAP, mobilization of nutrients from the pod wall to the seeds and growth have ceased.

In total, these data support a model in which mineral accumulation in the seeds is determined by the source strength of the pod wall and leaves and the timing of developmental transitions within these tissues. Beginning at pollination, the subtending leaf provides energy and nutrients to the developing pod. During early embryonic development (0–8 DAP), the majority of the dry matter and nutrients are partitioned to the pod wall, and this compartment accumulates the majority of translocated nutrients (Figure 6). As development continues, the export processes in the pod walls become more active, eventually leading to the attenuated mineral and dry matter accumulation in the pod walls. By 20 DAP, influx from the leaves to the pod walls and efflux from the pod walls to the seeds are equivalent for most minerals (Figure 6). Increased source activity of the pod wall is also associated with its decreased rate of growth, and following 20 DAP, the rate of dry mass and mineral accumulation in the pod wall decreases. Mineral accumulation in the seeds continues at a similar rate until 24–28 DAP, then begins to slow, such that by 32–36 DAP translocation to the seeds has ceased (Figure 6).

In our study, we observed genotypic variation in the timing of transitions between phases of accumulation and disparities in the overall uptake and translocation of specific minerals. Given that genotypic differences in growth and the patterns of mineral accumulation and loss from specific compartments did not result in significant differences in seed content, the combined effects of these different mechanisms may modulate seed mineral content. The recombinant inbred population generated by crossing these two ecotypes included lines that displayed significant variation in seed mineral content (Sankaran et al., 2009); thus, our data can be used to begin identifying traits in source and sink tissues that are associated with increased seed mineral levels. For instance, higher levels of seed K and Mg content were associated with the A17 allele of a specific genetic marker (Sankaran et al., 2009). Furthermore, there were few significant differences (i.e., at one or two time points) in the content of these minerals in the leaves and pod walls, and their patterns of accumulation were similar between ecotypes (Supplementary Presentations 2, 4). In the seeds, however, the genotypes diverged with respect to the timing of accumulation. In A17 seeds, these minerals tended to accumulate through 32 DAP, whereas in DZA seeds, accretion subsided by 28 DAP (Figure 4), suggesting that sustained activity of mineral delivery mechanisms through latter stages of development are key in determining the final content.

Our data also suggest that mechanisms controlling mineral translocation between organs modulate seed content. An area of particular interest is the contribution of processes occurring in the pod wall, as these relate to seed mineral content. As mentioned previously, accumulation of Mo in the pod walls was associated with lower Mo content per seed (Tables 2, 3). Furthermore, higher total seed mineral content per pod was observed in ecotype A17 (Table 4), and partitioning to the seeds was higher in this ecotype compared to DZA315.16 for most minerals (Figure 5C). Given that the mineral content in DZA pod walls was either equivalent or higher than their A17 counterparts, these data highlight the potential for achieving increased seed mineral content by increasing mobilization of minerals from the pod walls to the seeds. Remobilization of nutrients from the pod wall to the seeds may be mediated by specific transporters involved in either phloem loading or unloading (Bennett et al., 2011). As an example, the *gtr1-gtr2* double mutant of *Arabidopsis*, in which glucosinolate transporters 1 and 2 are knocked-out, displayed increased glucosinolate content in the silique and decreased glucosinolate content in the seeds (Nour-Eldin et al., 2012). This suggests that increasing expression of mineral transporters within the pod wall may then enhance partitioning of minerals into the seeds, providing an additional strategy for improving the nutritional quality of legume seeds.

Additional studies are required to identify the genes responsible for mediating mineral transport within the pod wall. Trait mapping has already isolated loci associated with variation in mineral content (Sankaran et al., 2009), and current projects are evaluating the contribution of specific genes to this process. Increasing the expression of these mediators will lead to enhanced mobilization from the pod wall to the seeds. Our findings also suggest that extending the duration of mineral transport through the latter stages of seed development will increase mineral content; therefore, understanding the mechanisms that control developmental processes in the source leaves and pod wall are also necessary for crop improvement.

## Author contributions

CG planned and performed experiments, analyzed data, and wrote and revised the manuscript. MG planned experiments, analyzed data, and revised the manuscript.

### Conflict of interest statement

The authors declare that the research was conducted in the absence of any commercial or financial relationships that could be construed as a potential conflict of interest.

## Footnotes

^1^Rasband, W. S. ImageJ. U. S. National Institutes of Health, Bethesda, Maryland, USA. Available online at: http://imagej.nih.gov/ij/.

## References

[B1] Benatti M. R. Yookongkaew N. Meetam M. Guo W. J. Punyasuk N. AbuQamar S.. (2014). Metallothionein deficiency impacts copper accumulation and redistribution in leaves and seeds of *Arabidopsis*. New Phytol. 202, 940–951. 10.1111/nph.12718 24635746

[B2] Bennett E. J. Roberts J. A. Wagstaff C. (2011). The role of the pod in seed development: strategies for manipulating yield. New Phytol. 190, 838–853. 10.1111/j.1469-8137.2011.03714.x 21507003

[B3] Blair M. W. (2013). Mineral biofortification strategies for food staples: the example of common bean. J. Agric. Food Chem. 61, 8287–8294. 10.1021/jf400774y 23848266

[B4] Broughton W. J. Hernandez G. Blair M. Beebe S. Gepts P. Vanderleyden J. (2003). Beans (*Phaseolus* spp.)—model food legumes. Plant Soil 252, 55–128. 10.1023/A:102414671061118055156

[B5] Burton J. W. Harlow C. Theil E. C. (1998). Evidence for reutilization of nodule iron in soybean seed development. J. Plant Nutr. 21, 913–927. 10.1080/01904169809365453

[B6] de Hoon M. J. Imoto S. Nolan J. Miyano S. (2004). Open source clustering software. Bioinformatics 20, 1453–1454. 10.1093/bioinformatics/bth078 14871861

[B7] Farnham M. W. Keinath A. P. Grusak M. A. (2011). Mineral concentration of broccoli florets in relation to year of cultivar release. Crop Sci. 51, 2721–2727. 10.2135/cropsci2010.09.0556

[B8] Gallardo K. Firnhaber C. Zuber H. Héricher D. Belghazi M. Henry C.. (2007). A combined proteome and transcriptome analysis of developing *Medicago truncatula* seeds: evidence for metabolic specialization of maternal and filial tissues. Mol. Cell. Proteomics 6, 2165–2179. 10.1074/mcp.M700171-MCP200 17848586

[B9] Hegelund J. N. Pedas P. Husted S. Schiller M. Schjoerring J. K. (2012). Zinc fluxes into developing barley grains: use of stable Zn isotopes to separate root uptake from remobilization in plants with contrasting Zn status. Plant Soil 361, 241–250. 10.1007/s11104-012-1272-x

[B10] Himelblau E. Amasino R. M. (2001). Nutrients mobilized from leaves of *Arabidopsis* thaliana during leaf senescence. J. Plant Physiol. 158, 1317–1323. 10.1078/0176-1617-00608

[B11] Hocking P. J. Pate J. S. (1977). Mobilization of minerals to developing seeds of legumes. Ann. Bot. 41, 1259–1278.

[B12] Marschner P. (ed.). (2012). Marschner's Mineral Nutrition of Higher Plants. 3rd Edn. San Diego: Academic Press.

[B13] Munier-Jolain N. Larmure A. Salon C. (2008). Determinism of carbon and nitrogen reserve accumulation in legume seeds. C. R. Biol. 331, 780–787. 10.1016/j.crvi.2008.07.020 18926492

[B14] Murgia I. Arosio P. Tarantino D. Soave C. (2012). Biofortification for combating “hidden hunger” for iron. Trends Plant Sci. 17, 47–55. 10.1016/j.tplants.2011.10.003 22093370

[B15] Nour-Eldin H. H. Andersen T. G. Burow M. Madsen S. R. Jørgensen M. E. Olsen C. E.. (2012). NRT/PTR transporters are essential for translocation of glucosinolate defence compounds to seeds. Nature 488, 531–534. 10.1038/nature11285 22864417

[B16] Pottier M. Masclaux-Daubresse C. Yoshimoto K. Thomine S. (2014). Autophagy as a possible mechanism for micronutrient remobilization from leaves to seeds. Front. Plant Sci. 5:11. 10.3389/fpls.2014.00011 24478789 PMC3900762

[B17] Saldanha A. J. (2004). Java Treeview—Extensible visualization of microarray data. Bioinformatics 20, 3246–3248. 10.1093/bioinformatics/bth349 15180930

[B18] Sankaran R. P. Grusak M. A. (2014). Whole shoot mineral partitioning and accumulation in pea (Pisum sativum). Front. Plant Sci. 5:149. 10.3389/fpls.2014.00149 24795736 PMC4006064

[B19] Sankaran R. P. Huguet T. Grusak M. A. (2009). Identification of QTL affecting seed mineral concentrations and content in the model legume *Medicago truncatula*. Theor. Appl. Genet. 119, 241–253. 10.1007/s00122-009-1033-2 19396421

[B20] Schiltz S. Munier-Jolain N. Jeudy C. Burstin J. Salon C. (2005). Dynamics of exogenous nitrogen partitioning and nitrogen remobilization from vegetative organs in pea revealed by 15N *in vivo* labeling throughout seed filling. Plant Physiol. 137, 1463–1473. 10.1104/pp.104.056713 15793068 PMC1088335

[B21] Uauy C. Distelfeld A. Fahima T. Blechl A. Dubcovsky J. (2006). A NAC Gene regulating senescence improves grain protein, zinc, and iron content in wheat. Science 314, 1298–1301. 10.1126/science.1133649 17124321 PMC4737439

[B22] Van de Velde W. Guerra J. C. De Keyser A. De Rycke R. Rombauts S. Maunoury N.. (2006). Aging in legume symbiosis. A molecular view on nodule senescence in *Medicago truncatula*. Plant Physiol. 141, 711–720. 10.1104/pp.106.078691 16648219 PMC1475454

[B23] Vaz Patto M. C. Amarowicz R. Aryee A. N. A. Boye J. I. Chung H.-J. Martín-Cabrejas M. (2014). Achievements and challenges in improving the nutritional quality of food legumes. Crit. Rev. Plant Sci. 34, 105–143. 10.1080/07352689.2014.897907

[B24] Wang H. L. Grusak M. A. (2005). Structure and development of *Medicago truncatula* pod wall and seed coat. Ann. Bot. 95, 737–747. 10.1093/aob/mci080 15703184 PMC4246729

[B25] Waters B. M. Chu H. H. Didonato R. J. Roberts L. A. Eisley R. B. Lahner B.. (2006). Mutations in *Arabidopsis* yellow stripe-like1 and yellow stripe-like3 reveal their roles in metal ion homeostasis and loading of metal ions in seeds. Plant Physiol. 141, 1446–1458. 10.1104/pp.106.082586 16815956 PMC1533956

[B26] Weber H. Borisjuk L. Wobus U. (2005). Molecular physiology of legume seed development. Annu. Rev. Plant Biol. 56, 253–279. 10.1146/annurev.arplant.56.032604.144201 15862096

[B27] Zheng L. Yamaji N. Yokosho K. Ma J. F. (2012). YSL16 Is a phloem-localized transporter of the copper-nicotianamine complex that is responsible for copper distribution in rice. Plant Cell 24, 3767–3782. 10.1105/tpc.112.103820 23012434 PMC3480301

[B28] Zuber H. Poignavent G. Le Signor C. Aimé D. Vieren E. Tadla C.. (2013). Legume adaptation to sulfur deficiency revealed by comparing nutrient allocation and seed traits in *Medicago truncatula*. Plant J. 76, 982–996. 10.1111/tpj.12350 24118112

