# Supplementary material for: Mineral accumulation in vegetative and reproductive tissues during seed development in Medicago truncatula
Source: Front Plant Sci. 2015 Aug 14;6:622. doi: 10.3389/fpls.2015.00622 (PMC4536387; doi:10.3389/fpls.2015.00622)
Supplement: Supplementary file 1 [file Table1.PDF]

**Mineral accumulation in vegetative and reproductive tissues during seed development in *Medicago truncatula***

**Christina B. Garcia and Michael A. Grusak***

*** Correspondence: mike.grusak@ars.usda.gov**

**Supplementary Table 1. Leaf mineral concentration.**

| | A17 | | | DZA315.16 | | |
|---|---|---|---|---|---|---|
| *Mineral* | *overall* | *Pollination* | *Maturity* | *overall* | *Pollination* | *Maturity* |
| Ca (mg/g DW) | - | 16.2 ± 0.6 | 27.7 ± 1.7 | + | 13.9 ± 0.8 | 33.1 ± 0.5 |
| Cu (µg/g DW) | + | 7.88 ± 0.51 | 4.61 ± 0.61 | + | 8.2 ± 0.3 | 5.1 ± 0.6 |
| Fe (µg/g DW) | - | 81.4 ± 14.4 | 67.5 ± 4.02 | - | 65.6 ± 4.1 | 92.9 ± 14.9 |
| K (mg/g DW) | - | 26.2 ± 1.3 | 34.3 ± 2.7 | + | 19.5 ± 1.1 | 27.2 ± 0.5 |
| Mg (mg/g DW) | + | 6.61 ± 0.35 | 10.5 ± 0.5 | + | 4.66 ± 0.28 | 10.3 ± 0.4 [1] |
| Mn (µg/g DW) | + | 15.3 ± 1.0 | 12.5 ± 1.37 | - | 28.1 ± 1.6 | 26.8 ± 3.7 |
| Mo (µg/g DW) | - | 37.9 ± 9.7 | 68.0 ± 27.1 | - | 15.9 ± 5.4 | 27.7 ± 8.4 |
| P (mg/g DW) | - | 5.68 ± 0.38 | 4.32 ± 0.32 | - | 4.36 ± 0.49 | 4.51 ± 0.39 |
| S (mg/g DW) | - | 3.77 ± 0.34 | 4.12 ± 0.27 | + | 2.87 ± 0.15 | 4.73 ± 0.38 |
| Zn (µg/g DW) | + | 17.1 ± 1.4 | 10.6 ± 1.2 | - | 19.1 ± 0.7 | 18.2 ± 0.1 |

Results of *a priori* statistical tests (*overall*), mineral concentration at baseline (leaf samples harvested from nodes on the day of flower pollination, *Pollination*), mineral concentration at pod maturity (*Maturity*), and results of pairwise comparisons between A17 and DZA315.16 at pollination (*P*) and maturity (*M*) are given. For overall analyses, leaves from each ecotype were harvested every four days beginning at pollination through pod maturity, and repeated measures ANOVA or Friedman's test was used to compare mineral concentrations at each time point. Minerals whose concentration changed significantly ($p<0.05$) are marked with (+); minerals whose concentration did not change significantly over time ($p>0.05$) are marked with (-). Average concentration ± standard error of the mean (SEM) of four samples is given.

[1]Denotes minerals with significant differences ($p<0.05$) in the pairwise comparison between the concentration at pollination and maturity by Tukey-Kramer testing (repeated measures ANOVA post hoc test).
