# Supplementary material for: Mineral accumulation in vegetative and reproductive tissues during seed development in Medicago truncatula
Source: Front Plant Sci. 2015 Aug 14;6:622. doi: 10.3389/fpls.2015.00622 (PMC4536387; doi:10.3389/fpls.2015.00622)
Supplement: Supplementary file 2 [file Table2.PDF]

Mineral accumulation in vegetative and reproductive tissues during seed development in *Medicago truncatula*

Christina B. Garcia and Michael A. Grusak*

*** Correspondence: mike.grusak@ars.usda.gov**

Supplementary Table 2. Pod wall mineral concentrations.

	A17			DZA315.16		
Mineral	*overall*	*8 DAP*	*Maturity*	*overall*	*8 DAP*	*Maturity*
Ca (mg/g DW)	-	13.4 ± 1.5	11.2 ± 0.68	-	18.8 ± 0.5	15.7 ± 0.6
Cu (µg/g DW)	+	6.18 ± 0.30	3.32 ± 0.04	+	11.5 ± 0.8	5.08 ± 0.47
Fe (µg/g DW)	+	*25.2 ± 6.4	9.06 ± 0.90	+	68.1 ± 12.9	26.4 ± 8.2
K (mg/g DW)	+	16.9 ± 1.3	12.7 ± 0.1	-	18.3 ± 0.5	14.7 ± 0.3
Mg (mg/g DW)	+	7.34 ± 0.74	4.21 ± 0.48	+	8.83 ± 0.46	5.81 ± 0.43
Mn (µg/g DW)	+	18.4 ± 1.1	10.5 ± 0.7	+	46.1 ± 5.3	38.5 ± 7.6
Mo (µg/g DW)	-	11.9 ± 2.6	9.68 ± 1.69	+	6.58 ± 1.46	20.8 ± 1.4
P (mg/g DW)	+	4.28 ± 0.21	1.47 ± 0.14	+	4.71 ± 0.06	2.5 ± 0.1
S (mg/g DW)	+	1.84 ± 0.09	0.774 ± 0.102	+	1.93 ± 0.07	1.18 ± 0.12
Zn (µg/g DW)	+	30.4 ± 4.4	9.55 ± 3.10	+	42.2 ± 5.2	22.9 ± 3.3

Results of *a priori* statistical tests (*overall*), mineral concentration from samples harvested at 8 days (or 12 days, indicated by *) after flower pollination (*8 DAP*), mineral concentration at pod maturity (*Maturity*), and results of pairwise comparisons between A17 and DZA315.16 at 8 DAP (*8*) and maturity (*M*) are given. For overall analyses, pods were harvested every four days starting at 8 DAP through pod maturity, and repeated measures ANOVA or Friedman's test was used to compare mineral concentrations at each time point. Minerals whose concentration changed significantly ($p<0.05$) at any time point from 8 DAP through pod maturity are marked with (+); minerals whose concentration did not change significantly over time ($p>0.05$) are marked with (-). Average concentration ± standard error of the mean (SEM) of four samples is given.
