# Supplementary material for: Mineral accumulation in vegetative and reproductive tissues during seed development in Medicago truncatula
Source: Front Plant Sci. 2015 Aug 14;6:622. doi: 10.3389/fpls.2015.00622 (PMC4536387; doi:10.3389/fpls.2015.00622)
Supplement: Supplementary file 3 [file Table3.PDF]

Mineral accumulation in vegetative and reproductive tissues during seed development in *Medicago truncatula*

Christina B. Garcia and Michael A. Grusak*

*** Correspondence: mike.grusak@ars.usda.gov**

Supplementary Table 3. Seed mineral concentrations.

	A17			DZA315.16		
Mineral	*overall*	*20 DAP*	*Maturity*	*overall*	*20 DAP*	*Maturity*
Ca (mg/g DW)	+	2.01 ± 0.03	1.48 ± 0.06	+	2.87 ± 0.21	1.42 ± 0.08
Cu (µg/g DW)	-	8.85 ± 0.36	11.9 ± 0.7	-	19.0 ± 1.6	27.4 ± 1.2
Fe (µg/g DW)	+	96.6 ± 5.6	105 ± 3	+	140 ± 23	154 ± 21
K (mg/g DW)	-	13.6 ± 0.1	11.5 ± 0.1	-	16.1 ± 0.2	12.1 ± 0.2
Mg (mg/g DW)	-	3.15 ± 0.13	3.14 ± 0.03	-	3.63 ± 0.10	3.15 ± 0.02
Mn (µg/g DW)	+	23.4 ± 1.0	19.4 ± 0.9	+	28.3 ± 0.7	20.4 ± 0.5
Mo (µg/g DW)	+	38.8 ± 1.1	64.4 ± 1.0	+	27.5 ± 2.6	44.5 ± 1.7
P (mg/g DW)	-	7.33 ± 0.08	9.75 ± 0.10	+	6.75 ± 0.03	10.3 ± 0.1
S (mg/g DW)	+	3.08 ± 0.18	3.76 ± 0.12	+	3.08 ± 0.15	3.63 ± 0.03
Zn (µg/g DW)	-	57.4 ± 2.6	58.0 ± 3.0	-	81.0 ± 7.1	74.3 ± 7.7

Results of a priori statistical tests (*overall*), mineral concentration at baseline (seeds samples harvested 20 days after flower pollination, *20 DAP*), mineral concentration at pod maturity (*Maturity*), and results of pairwise comparisons between A17 and DZA315.16 at 20 DAP (*20*) and maturity (*M*) are given. For overall analyses, seeds from each ecotype were harvested every four days from 20 DAP through pod maturity, and repeated measures ANOVA or Friedman's test was used to compare mineral concentrations at each time point. Minerals whose concentration changed significantly ($p<0.05$) at any time point from 20 DAP through pod maturity are marked with (+); minerals whose concentration did not change significantly over time ($p>0.05$) are marked with (-). Average concentration ± standard error of the mean (SEM) of four samples is given.
