# Supplementary material for: Mineral accumulation in vegetative and reproductive tissues during seed development in Medicago truncatula
Source: Front Plant Sci. 2015 Aug 14;6:622. doi: 10.3389/fpls.2015.00622 (PMC4536387; doi:10.3389/fpls.2015.00622)
Supplement: Supplementary file 4 [file Presentation1.PDF]

Supplementary Presentation 1

Mineral accumulation in vegetative and reproductive tissues during seed development in *Medicago truncatula*

Christina B. Garcia and Michael A. Grusak*

*** Correspondence:** Michael A. Grusak: mike.grusak@ars.usda.gov

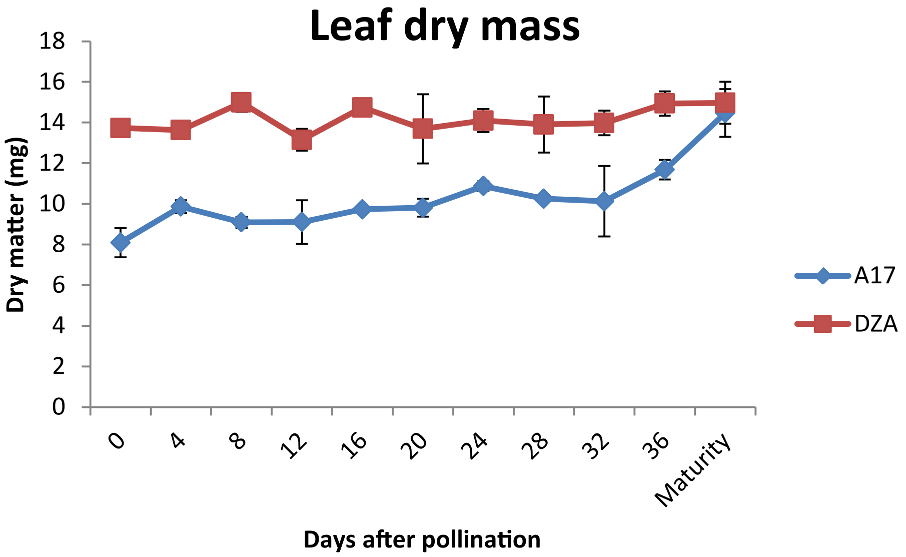

Supplementary Presentation 1. Average leaf dry mass. Leaves were harvested from from A17 and DZA315.16 plants at each time point beginning at pollination (0) and continuing through pod maturity (Maturity). The dry masses of multiple leaves harvested from independently grown plants were averaged. Error bars denote SEM for each time point.
