# Supplementary material for: Mineral accumulation in vegetative and reproductive tissues during seed development in Medicago truncatula
Source: Front Plant Sci. 2015 Aug 14;6:622. doi: 10.3389/fpls.2015.00622 (PMC4536387; doi:10.3389/fpls.2015.00622)
Supplement: Supplementary file 9 [file Presentation6.PDF]

Supplementary Presentation 6

Mineral accumulation in vegetative and reproductive tissues during seed development in *Medicago truncatula*

Christina B. Garcia and Michael A. Grusak*

* **Correspondence:** Michael A. Grusak: mike.grusak@ars.usda.gov

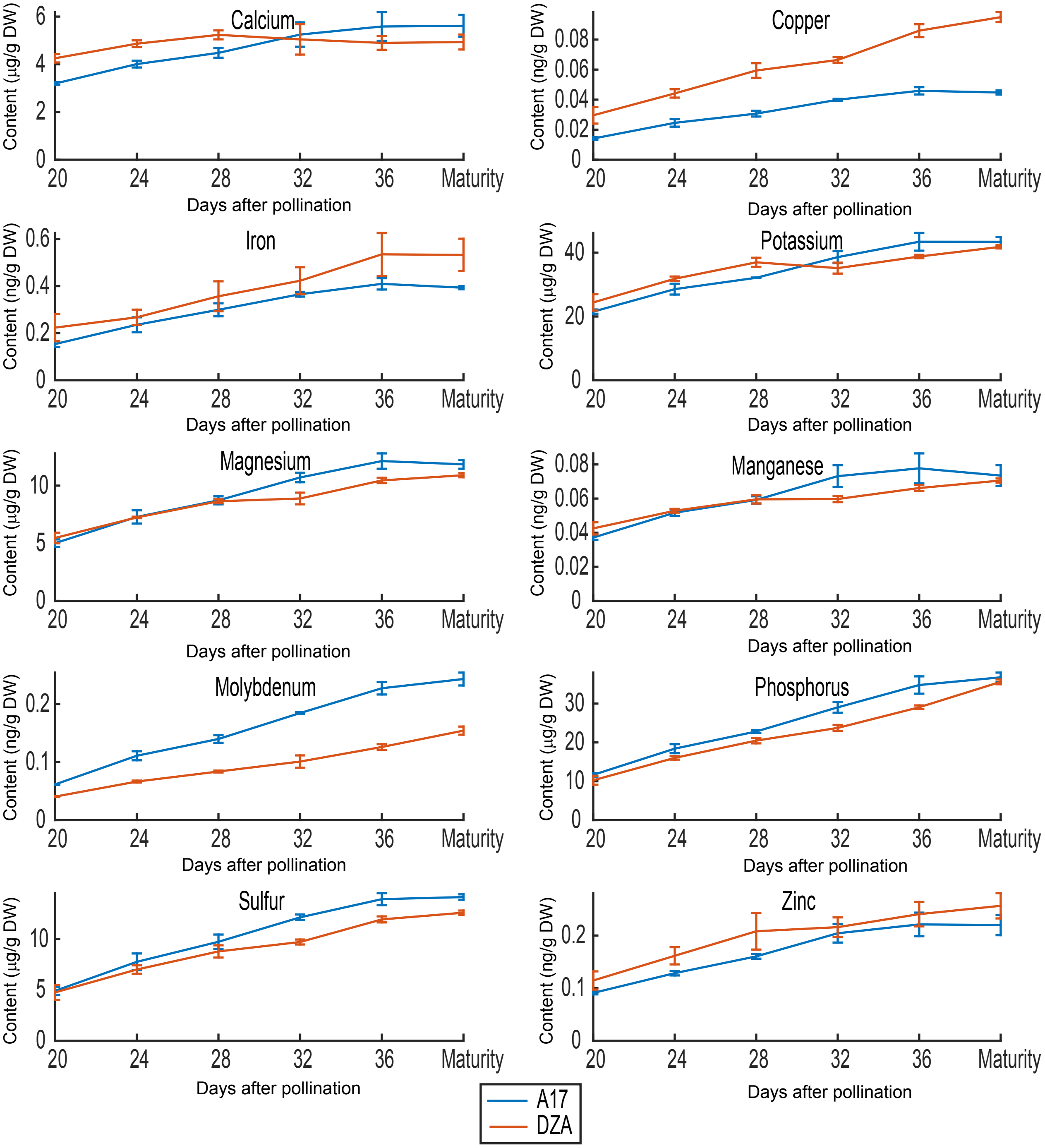

Supplementary Presentation 6. Average seed mineral content during reproductive growth. Mineral content was calculated as described in Materials and Methods section. The average of four samples harvested at each time point for A17 (blue line) and DZA315.16 (red line) is depicted. Error bars display the SEM.
