# Supplementary material for: Mineral accumulation in vegetative and reproductive tissues during seed development in Medicago truncatula
Source: Front Plant Sci. 2015 Aug 14;6:622. doi: 10.3389/fpls.2015.00622 (PMC4536387; doi:10.3389/fpls.2015.00622)
Supplement: Supplementary file 10 [file Presentation7.PDF]

Supplementary Presentation 7

Mineral accumulation in vegetative and reproductive tissues during seed development in *Medicago truncatula*

Christina B. Garcia and Michael A. Grusak*

*** Correspondence:** Michael A. Grusak: mike.grusak@ars.usda.gov

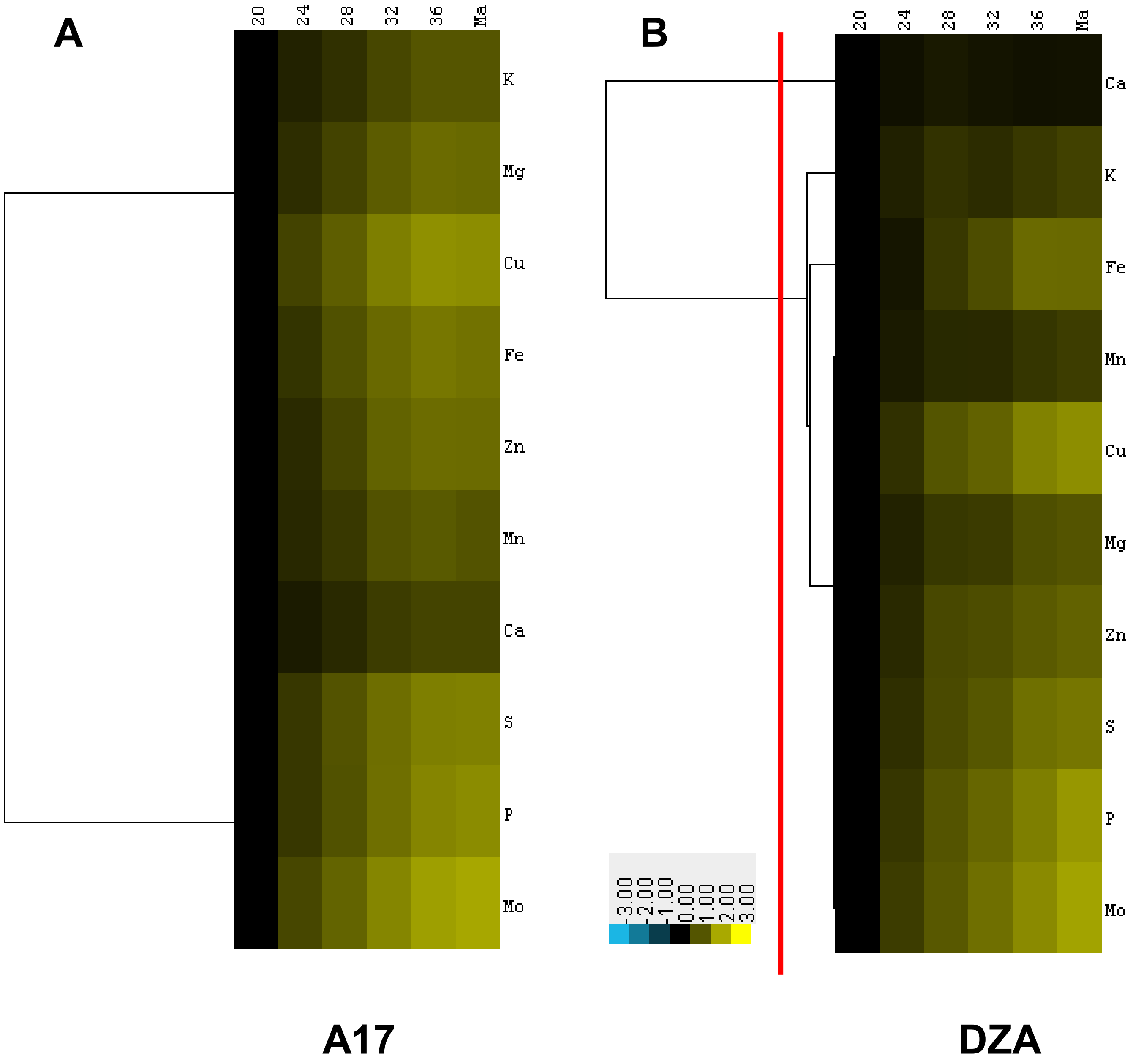

Supplementary Presentation 7. Hierarchical clustering of minerals in the seeds of A17 and DZA315.16 by content change over time. Fold differences between the content at each time point and the content at 0 DAP were calculated and transformed to the log2 scale. Hierarchical clustering was performed using Cluster 3.0, and heat maps and dendrograms were generated using Java TreeView for A17 (A) and DZA315.16 (B). A) All minerals clustered together, correlation = 1.0. B) Vertical red line denotes the correlation cut-off (correlation > 0.8) where clusters that connected to the right of the red line were considered related, and clusters connected to the left of the red line were considered unrelated.
